# Nucleic Acid-Based Therapy Approaches for Huntington's Disease

**DOI:** 10.1155/2012/358370

**Published:** 2012-01-11

**Authors:** Tatyana Vagner, Deborah Young, Alexandre Mouravlev

**Affiliations:** ^1^Department of Molecular Medicine and Pathology and Centre for Brain Research, The University of Auckland, Auckland 1142, New Zealand; ^2^Department of Pharmacology and Clinical Pharmacology and Centre for Brain Research, The University of Auckland, Auckland 1142, New Zealand

## Abstract

Huntington's disease (HD) is caused by a dominant mutation that results in an unstable expansion of a CAG repeat in the huntingtin gene leading to a toxic gain of function in huntingtin protein which causes massive neurodegeneration mainly in the striatum and clinical symptoms associated with the disease. Since the mutation has multiple effects in the cell and the precise mechanism of the disease remains to be elucidated, gene therapy approaches have been developed that intervene in different aspects of the condition. These approaches include increasing expression of growth factors, decreasing levels of mutant huntingtin, and restoring cell metabolism and transcriptional balance. The aim of this paper is to outline the nucleic acid-based therapeutic strategies that have been tested to date.

## 1. Introduction

Huntington's disease (HD is an inherited autosomal-dominant disorder characterised by loss of motor control, cognitive decline, psychiatric disturbances, and dementia, which progresses towards death within approximately 20 years of disease onset [1]. It is caused by an expansion of a CAG repeat in the huntingtin gene (*Htt*) that results in synthesis of an aberrant polyglutamine tract in huntingtin protein (HTT) and leads to neuronal dysfunction and neurodegeneration [2]. GABAergic medium-sized spiny neurons in the striatum are found to be most profoundly affected [3]; neuronal loss in the cerebral cortex, hippocampus, hypothalamus, substantia nigra, and in other brain structures has also been reported [4].

The mechanism by which the mutant huntingtin (m*Htt*) causes HD is still poorly understood. Normal HTT has been shown to have multiple distinct functions in cells, including antiapoptotic activity [5–7], roles in vesicular transport [8–11], neuronal gene transcription regulation [12, 13], and control of synaptic transmission [14, 15]. Consequently, the expression of long glutamine stretches either in the context of an N-terminal fragment or full-length HTT disrupts a wide variety of biological functions in cellular as well as animal models.

To date, many animal models have been developed that closely mimic HD symptoms or pathology. Rodents (mouse, rat) and nonhuman primates have been used most extensively to test HD gene therapy strategies. In general, HD animal models can be divided into chemically induced and genetic models. The chemically induced HD models include excitotoxic lesion models (glutamate-, kainic acid-, quinolinic acid- (QA-) induced) [16–19] and mitochondrial dysfunction models (3-nitroprionic acid (3-NP), malonate induced) [20–22]. Since HD is known to be a hereditary disorder, genetic models aim to mimic the molecular pathogenesis of HD more closely than that of chemical lesion models. The main types of genetic HD models are represented by transgenic (bearing the full-length or N-terminal fragment of *Htt* in their genome) [23, 24] and knock-in (expressing pathological length CAG repeat inserted into endogenous *Htt* gene) [25–27] animals.

The majority of therapeutics currently used to treat HD are designed to ameliorate the symptomatology of the condition, that is, psychiatric agents for the control of behavioural symptoms, motor sedatives, cognitive enhancers [28–30], and neuroprotective agents [31–37]. These drugs have limited benefits and do not address the disease progression. Meanwhile, gene therapy provides promising approaches in treating HD, and sidesteps the need to understand how expression of the expanded CAG repeat in *Htt* causes the disease. These can be broadly classed into strategies involving (i) increasing expression levels of growth factors, (ii) decreasing levels of mutant HTT, and (iii) restoring cell metabolism and transcriptional balance.

## 2. Neuroprotective and Neuroregenerative Approaches for HD

Since the behavioural phenotypes in HD arise from a progressive loss of mainly striatal and cortical neurons, neuroprotection and neurorestoration are one of the major gene therapy approaches being developed. Early gene therapy strategies for HD focused on the delivery of neurotrophic factor genes as a direct means for protecting vulnerable striatal neurons against mutant HTT-mediated toxicity. Other alternatives have included delivery of molecules aimed at directing neurogenesis—the production of new adult neurons to replace neurons lost in the disease [38].

Neurotrophic factors prevent cell death in degenerative processes and enhance growth and function of neurons. Several neurotrophic factors have shown promise as therapeutic agents in cell lines and animal models. Nerve growth factor (NGF) was the first trophic factor evaluated in an excitotoxic rodent model of HD. However, the potent protection from degeneration with nearly two-thirds of the neurons in the QA-injured striatum rescued was found only when NGF was delivered into striatum by *ex vivo* modified cell grafts [39–42], but not when it was infused directly [43, 44]. The infusions provided neuroprotection to the cholinergic neuron population within the striatum while GABAergic neurons that are preferentially vulnerable in HD were not protected. Alternative growth factor candidates have also been evaluated and beneficial effects were found with delivery of neurturin (NTN) and glial cell line-derived neurotrophic factor (GDNF). Adenoassociated virus- (AAV-) delivered NTN reduced the extent of striatal neuronal cell death (24% cell loss in the NTN-treated group versus *≈*43% in control groups), and attenuated functional disability associated with striatal lesions produced by 3-NP infusion. Locomotor coordination as assessed by performance on the accelerating rotarod of animals expressing the NTN-producing transgene was similar to those of unlesioned animals [45]. Similarly, AAV-mediated delivery of GDNF in rat [46] and mouse [47] models of HD has been shown to provide structural and functional neuroprotection, promoting approximately 70% neuron survival as compared to untreated animals and improving behavioural performances at the platform and the hind limb clasping tests to a near normal level. *Ex vivo* delivery of GDNF also reduced neuronal death and maintained motor functions, although to a lesser extent. This was attributable to lower levels of transgene expression compared to those achieved by direct *in vivo *viral-mediated gene transfer, also, GDNF expression appeared limited to the area immediately surrounding the transplant core, and only 1% of total cells injected survived 3 months after transplantation [46]. Interestingly, intrastriatal lentivirus-mediated delivery of GDNF did not ameliorate neurological and behavioural impairments in the R6/2 transgenic mice model of HD [47]. This might be due to the incorrect timing of the treatment administration: GDNF overexpression might have been initiated too late in the course of the disease, when mutant huntingtin may already have triggered an irreversible pathogenetic process. Also, dependence of the neuroprotective effect on the particular mechanism of cell death in transgenic versus chemical HD models may be possible.

Encouraging results were obtained following ciliary neurotrophic factor (CNTF) infusion. Although lentiviral-mediated overexpression of CNTF in the striatum of YAC72 mice, a genetic mouse model of HD, reduced hyperactivity in 5 and 8 month old mice, no differences in rotarod performance or feet-clasping was found compared to controls [48]. In excitotoxic rodent and primate models, CNTF provided significant neuroprotection when delivered by means of genetically modified baby hamster kidney (BHK) cell grafts encapsulated in semipermeable membrane [49], osmotic minipump [50], lentiviral [51] and adenoviral [52] transfer represented by a 52–64**% **reduction in lesion volumes and twice as big cell survival rate versus control groups. The experiments using the first technique showed either no difference or only a slight difference in neuron density between CNTF-treated and unlesioned brains, except for the lateral caudate where the decrease in density was 11% for NeuN-immunoreactive and 18% for calbindin-immunoreactive neurons. CNTF-expressing BHK cell grafts were also employed in a phase I clinical study involving a small number of HD patients. Whilst no side effects were observed over a period of 2 years, no clinical benefit was observed in these patients, most likely due to the low amounts of CNTF release produced by many of the capsules and low survival of the encapsulated cells [53].

Much research on HD has focused on brain-derived neurotrophic factor (BDNF), which is considered to be the main candidate for neuroprotective therapeutic strategies for HD. BDNF has been shown to modulate the onset and severity of motor and cognitive functions in HD mouse models [54–56]. Mutant huntingtin reduces the transcriptional activity of the BDNF promoters, thus reducing the synthesis of BDNF protein in the cerebral cortex [12, 13], where approximately 95% of striatal BDNF is produced before being transported to its striatal targets via the corticostriatal afferents. It has also been reported that HD patients have lowered levels of BDNF in the cerebral cortex and striatum [57], and BDNF levels are also decreased in many mouse and cell models of the disease [58]. Adenoviral (Ad) vector-mediated delivery of BDNF reduced the size of QA-induced lesions in rats by one half, with 64% of medium spiny projection neurons surviving in Ad/BDNF-treated animals compared to 46% of those in controls [59]. AAV-mediated delivery of BDNF in similar excitotoxic rat model also provided neuroprotection to vulnerable striatal neurons (71–78% versus 48–54% of preserved cells as shown by NeuN and calbindin immunoreactivity in BDNF-treated versus untreated striata, resp.) [60] and ameliorated motor dysfunction in tests designed to show hemispherical imbalances in brain function resultant of unilateral QA lesioning [61]. In contrast, neither *ex vivo* BDNF gene delivery [41, 62], nor direct BDNF protein infusion [50] has proved efficient in preventing the loss of striatal projection neurons following lesioning, presumably as the dosage of BDNF delivered may not have been sufficient to provide neuroprotection.

Although increasing BDNF expression by means of viral vectors has led to encouraging results, a number of issues still remain to be resolved, as excess expression of the BDNF transgene can have a deleterious effect on neuronal circuits and learning and memory [63], and some of the vectors are toxic *per se* and can cause tumour formation due to accidental insertional mutagenesis [64]. Furthermore, the questions of timing relative to intervention in the disease process, and anatomical location with respect to administration of the vector, must be addressed before applying the approach in patients, as transport of a transgene in axonal tracts could lead to unexpected side effects [65, 66].

## 3. Therapeutic Strategies Targeting Mutant Huntingtin

More recently, the development of effective gene silencing approaches using RNA interference technology has led to evaluation of strategies aimed at selectively reducing mutant *Htt* expression. This is an attractive approach to therapy as it sidesteps the requirement to understand the mechanism by which mutant *Htt* causes the disease pathology. The therapeutic promise of this direct approach is underpinned by a pivotal study demonstrating that repression of m*Htt* expression in a conditional mouse model could reverse the pathological features of the disease including formation of neuronal inclusions and abnormal motor behaviour [67]. Thus decreasing abnormal m*Htt* load using this approach during the disease course might facilitate better protein clearance by affected neurons and allow neurons to normalise changes induced by m*Htt*. Moreover, the ability to instigate treatments prior to onset of disease symptoms that is afforded by genetic screening for individuals that have inherited the HD mutation allows greater opportunity to make a significant impact on disease progression, when neuronal dysfunction might be prevalent but significant neurodegeneration and depletion of cortical and striatal neuron populations are yet to take place.

Modified antisense short nucleotide technology provides fascinating opportunities for development of nucleic acid-based therapeutics. Peptide nucleic acid (PNA) peptide conjugates and locked nucleic acid (LNA) oligomers were found to be potent and allele-selective inhibitors of mutant *Htt* expression in HD human cell lines. PNAs are DNA/RNA mimics with an uncharged amide backbone, that increases the affinity of PNA hybridization and facilitates recognition of RNA target, and LNA is an RNA analogue that contains a methylene bridge between the 2′-oxygen and 4′-carbon of the ribose, which reduces the conformational flexibility of the ribose and confers outstanding affinity to complementary hybridization. A comparison in the same model cell line revealed that inhibition by PNAs and LNAs that target CAG repeats is more effective than inhibition by an siRNA that targets a deletion polymorphism [68].

Early RNA interference-based approaches involved targeted knockdown of m*Htt* transcripts using species-specific short hairpin (shRNA) or short-interfering RNA (siRNA) in HD transgenic mouse models. Sequence differences between mouse host and human genes allowed allele-specific silencing of the pathogenic human *Htt* transcripts in the setting of preserved expression of endogenous *Htt*. Intraventricular infusions of lipid-encapsulated [69] or intrastriatal injections of cholesterol-conjugated [70] siRNAs effectively silenced mutant huntingtin transcripts by *≈*70% leading to a 56–66% reduction in HD protein levels. As a consequence, numbers and the size of intranuclear inclusions were decreased, and R6/2 transgenic mice and mice with the AAV-mediated *Htt *expression, respectively, showed improved performance in motor tests including beam walking and an accelerating rotarod.

One of the major hurdles for gene silencing is effective delivery of siRNA sequences to affected cells. The chronic nature of this disease suggests that continuous and long-term expression of siRNA will be required. This can be achieved by chronic infusion but whether lifelong infusion can be tolerated is unclear. An alternative approach is to use viral vectors to achieve long-term expression of shRNA molecules. AAV-mediated brain delivery of shRNA directed against human mutant *Htt* decreased mutant huntingtin mRNA and protein levels by 51–78% and 28–50%, respectively, reduced numbers of aberrant nuclear inclusions, and improved disease-associated behaviours such as the feet-clasping phenotype in HD mice and rotarod performance as well as spontaneous exploratory forepaw use in rats [71–73]. In the context of a lentiviral vector gene delivery system, shRNA targeted to the human *Htt* mRNA reduced *Htt* mRNA levels by more than 80% and almost completely prevented loss of dopamine and cAMP-regulated phosphoprotein-32 (DARPP-32) expression, and restored deficits in striatal glucose metabolism and mitochondrial complex II activity in a rat HD model. Expression of shRNA after appearance of HD pathology also produced a drastic reduction in the lesion size, associated with a partial clearance of HTT inclusions [74].

While the results of these proof-of-concept studies are encouraging, one challenge in extending this concept to human HD subjects is the potential requirement to develop approaches that selectively silence the mutant allele whilst leaving the normal allele intact. Normal huntingtin has roles in axonal guidance, cAMP signaling, long-term potentiation/depression, and calcium and glutamate signaling, raising concerns about potential toxicity resulting from loss of huntingtin, function [58]. Moreover HD gene knockout in mice causes developmental defects and embryonic lethality [75–77]. However, humans that are homozygote for the HD mutation [78, 79] and knock-in mice [33, 80, 81] do exist, despite expressing no normal huntingtin, suggesting knockdown of normal *Htt* might be tolerated to some extent in the adult brain. To determine whether silencing of the normal allele might exacerbate mHtt pathology, Drouet et al. [74] and Boudreau et al. [82] evaluated the effect of knockdown sequences that would attenuate expression of both mutant and normal *Htt* alleles. Lentiviral vector-mediated expression of a pathogenic human htt171-82Q on a background of reduced wild-type rat *Htt* levels did not produce any differences in GABAergic neuron survival or HTT inclusion load compared to rats that received the htt171-82Q alone suggesting partial inactivation of the endogenous rat allele did not increase the vulnerability of striatal neurons to m*Htt*. Moreover, coincident nonallele-specific silencing of m*Htt* and wild-type *Htt* is well tolerated in mice, with minimal signs of toxicity for up to 9 months [74]. However in the context of an AAV vector system, shRNAs have been associated with toxicity, potentially due to saturation of cellular RNAi processing mechanisms [82, 83] which may limit usage of this technology. Interestingly, placing the identical siRNA sequences into artificial miRNA backbones mitigates the toxic effects markedly [82, 84]. Moreover, switching the RNA interference mechanism toward that used by microRNAs (miRNA) by introducing one or more mismatched bases into these duplex RNAs might allow preferential silencing of the mutant allele over the normal allele [85]. Transgenic HD-171-82Q mice that received intrastriatal injections of an AAV vector expressing an miRNA sequence (mi2.4) that silences both mutant and wild-type mouse *Htt* mRNAs by *≈*60% showed significant improvements in rotarod performance as compared to control-treated HD171-82Q mice. Moreover, despite AAV1-mi2.4 failing to normalise the weight loss observed in these animals, survival rates of these mice were also increased [82]. 

An alternative approach might also involve targeting disease-allele-linked small nucleotide polymorphisms (SNPs) [86] or deletion polymorphisms [87]. This provides the opportunity to develop reagents selectively silencing the disease-causing allele. Many SNPs are prevalent on the m*Htt* transcript [88], and it has been suggested that only 5 duplex siRNAs would be sufficient to treat 75% of patients with HD, although the need to develop several reagents may be a complicating factor [89]. It is also unclear whether duplex RNAs will be able to achieve sufficient allele selectivity and potency in a therapeutic setting.

While these results are encouraging and suggest stringent targeted knockdown of the mutant allele may not be necessary, these studies have also reported significant transcriptomic changes in *Htt*-related molecular pathways when expression of the wild-type huntingtin is inhibited [74, 82]. Boudreau et al. [82] compared datasets of transcripts differentially expressed by >2-fold following knockdown of normal *Htt* in mouse striatum with a dataset obtained from early grade HD patient caudate-putamen relative to their respective controls. Ninety-two genes were found to be altered in both gene sets. Interestingly, 41 of these showed changes in expression level in the same direction and were enriched in pathways involving developmental regulation of gene transcription. Fifty-one other genes that were common to both gene sets changed in the opposite direction and were enriched for proteins involved in ion transport and synaptic transmission, many of which are downregulated in human HD. These authors propose that these transcriptional changes might result from toxic gain of function aspect and that knocking down both mutant and wild-type *Htt* might revert these changes. However, a recent and extensive analysis of gene expression changes altered by *Htt* deficiency compared with the effect of the gain of function conferred by increasing CAG repeat length suggests that it is important to understand the biological processes affected by the lowering of normal *Htt* expression [90]. Panels of mouse embryonic stem (ES) cell lines engineered to express a full-length huntingtin mouse homolog (Hdh) with a knock-in of CAG repeats of increasing lengths were used to distinguish between gene expression changes conferred by the CAG expansion to that produced by loss of the endogenous* Htt* allele (Hdh null). Microarray comparison between a gene set whose expression was continuously altered with increasing CAG length with that of a huntingtin-null gene set showed that there was virtually no overlap in these gene sets suggesting the CAG expansion confers a simple gain of a novel function as opposed to a mixed gain of function/loss of function mechanism. Although the molecular responses are quite distinct, the gene expression changes result in varying degrees of interconnectedness at the network level. For example, pathways involved in the energy network such as carbohydrate metabolism/glycolysis are more prominently affected by CAG repeat length whereas oxidative respiration and tricarboxylic acid cycle pathways are more affected by huntingtin deficiency [90]. These authors suggest that treatments aimed at lowering the expression of the expanded CAG allele might instead exacerbate the physiologic effects of the expanded CAG repeat and thus gaining further insight into the biological processes affected by the lack of normal huntingtin is important. Thus selectively reducing expression of the disease-causing trigger without affecting the normal allele would be a preferable approach.

An alternative to knocking down *Htt* mRNA expression involves boosting the capacity of the cell to lower amounts of mutant huntingtin protein. The neurodegenerative and motor, cognitive, and psychiatric symptoms of HD typically manifest in midlife suggesting that in the presymptomatic phase, neurons are able to cope to some extent with the expression of mutant huntingtin protein. In a conditional transgenic mouse model of HD, blockade of m*Htt* fragment expression in symptomatic mice leads to a disappearance of inclusions and an improvement in behavioural phenotypes, demonstrating that continuous production of the mutant protein is required to maintain the disease process and raising the possibility that HD may be reversible [67]. The half-life and clearance of normal and mutant HTT has not been studied in detail, however, enhancing the activity of molecular chaperones can promote refolding of misfolded proteins. For example, overexpression of one or both of the chaperones HSP104 and HSP27 has been shown to suppress mutant HTT-mediated neurotoxicity in a rat model of HD, promoting 59% neuronal survival *in vitro* and reducing striatal lesion by 35–65% *in vivo* [91]. Another therapeutic strategy uses intracellular antibodies (intrabodies) to target huntingtin. The intrabodies are recombinant antibodies maintaining the diversity, high specificity, and high affinity to the target site, characteristics of traditional antibodies, but they are smaller in size and have been genetically engineered to cross the blood-brain barrier and to function in the intracellular environment [92]. An intrabody approach has been assessed in various mouse models of HD [93]. It improved motor performance in the rotarod, beam crossing, climbing, and feet-clasping tests, and strongly ameliorated neuropathology including decreased numbers of striatal aggregates and a 2–2.5-fold reduction in lesion size in the intrabody-treated animals compared to untreated controls. In addition, G-rich oligonucleotide-based techniques that inhibit mutant HTT aggregation might also be of therapeutic value. A 20-mer, all G-oligonucleotide (HDG) capable of adopting a certain conformation, has been shown to block aggregation of a fusion protein that contained an N-fragment of huntingtin bearing an aberrant polyglutamine tract [94].

## 4. Strategies Promoting Cell Metabolism and Restoring Transcriptional Balance

An important aspect of HD pathogenesis is the impact of the mutant protein on mitochondrial function and cellular bioenergetics. Reduced glucose utilisation and activities of complexes of the electron transporter chain in the striatum of advanced-grade HD subjects suggests a general metabolic deficit in HD patients [95–98]. Additionally, the sensitivity of medium spiny neurons to mitochondrial poisons such as 3-NP suggests the disease is influenced by altered mitochondrial function [99]. Mutant HTT has direct and/or indirect effects on mitochondria, compromising cellular energy production and respiration that leads to a reduction of the intracellular level of ATP, thus promoting apoptosis, oxidative stress and susceptibility to excitotoxicity [100, 101].

Therapeutic agents that aim to ameliorate the cellular energy deficits by enhancing energy production and improvement of mitochondrial function in HD are neuroprotective. Lentiviral-mediated overexpression of two subunits of the succinate dehydrogenase (SDH) enzyme, the main component of mitochondrial complex II, restores the membrane potential and blocks neuronal death induced by mutant huntingtin in murine cell model of HD [102]. Another key target of mutant huntingtin is peroxisome proliferator-activated receptor gamma coactivator-1 *α* (PGC-1*α*), a transcriptional coactivator that regulates expression of genes involved in mitochondrial biogenesis and oxidative phosphorylation [103]. PGC-1*α* expression levels are specifically reduced in the caudate-putamen, the first region affected in HD, in presymptomatic postmortem HD cases. Moreover, a differential cellular expression pattern of PGC-1*α* is found in the caudate-putamen, with decreased expression in the vulnerable medium spiny neurons but upregulation in nNOS-immunoreactive interneurons which are typically spared in the disease [104, 105]. Decreased PGC-1*α* expression is due to m*Htt*-mediated repression of PGC-1*α* transcription via interference of CREB/TAF4-dependent regulation of PGC-1*α* gene expression. Inhibition of PGC1a expression leads to altered expression of genes involved in energy metabolism and diminished capacity of vulnerable neurons in response to energy demands in HD [105]. Cui et al. [105] determined whether genetic overexpression of PGC-1*α* could protect against m*Htt*-induced mitochondrial dysfunction and striatal toxicity. Direct administration of lentiviral vector expressing PGC-1*α* into the striatum of R6/2 transgenic HD mice completely prevented neuronal atrophy, complementing *in vitro* data showing PGC-1*α* overexpression reversed mitochondrial dysfunction in mutant STHdhQ111 cells, and abrogated the toxicity of m*Htt* in transfected primary striatal neurons, potentially through inducing the expression of genes encoding reactive oxygen species defence enzymes including Cu/Zn superoxide dismutase (SOD1), manganese SOD (SOD2), catalase, and glutathione peroxidase [105, 106]. This is in line with findings showing overexpression of SOD1, and molecular chaperones Hsp40 or Hsp70 in mutant HTT expressing mouse cells, prevents oxidative stress-induced proteasomal malfunction, mutant huntingtin aggregation, and cell death [107]. Whether PGC-1*α* overexpression results in improved survival and motor performance in the R6/2 mice remains to be confirmed. These results suggest that stimulation of pathways involved in energy metabolism controlled by PGC-1*α* by pharmacological or genetic means could provide potential clinical benefit at early stages of HD.

Huntingtin can interact with a number of transcription factors, for example, cAMP-response element binding (CREB) binding protein [108, 109], TATA-binding protein (TBP) [110], Sp1 [111], and p53 [109, 112], and consequently, transcriptional dysregulation occurs in the presence of mutant huntingtin [113]. One of the key genes, whose function is impaired in HD, is repressor element 1 silencing transcription factor (REST), a global repressor of neuronal gene expression, including BDNF. Disruption of REST target gene expression might be an early molecular event in HD [114] and thus attenuation of REST binding during early disease stages could be of therapeutic benefit. The employment of double-stranded oligodeoxynucleotide decoys corresponding to the DNA-binding element of the REST has been shown to abrogate its transcriptional activity and REST-mediated epigenetic repression rescuing levels of its target genes' mRNA and protein in the cell model of HD [115].

## 5. Current Challenges in Development of Gene Therapy for HD

Despite significant achievements in nucleic acid-based gene therapy for HD, a range of technical problems is needed to be solved and questions to be answered. Each strategy described has specific disadvantages. Though many studies have proven that gene therapy approaches employing antisense oligonucleotides represent an exciting therapeutic possibility, there are certain issues that remain to be addressed. These include the choice of cellular targets, the stability of gene silencing, as well as side effects, such as altering off-target gene expression, induction of cellular immune response, and interfering with endogenous mRNA silencing systems. There are also problems associated with viral delivery of therapeutic genes. Transgene expression can be difficult to standardize and regulate, and some of the vectors can be toxic *per se* and/or cause insertional mutagenesis. The incorporation of a regulatory systems such as the *tet* system might increase the safety profile of such a treatment approach. Moreover, although studies using viral vectors have shown sustained gene expression for many years, maintaining long-term expression is still a current challenge.

Apart from these particular impediments, there are basic issues typical of all strategies, such as invasiveness of delivery methods associated with the direct administration of therapeutics into the brain due to the presence of blood-brain barrier, restriction of therapeutics distribution only to the area adjacent to the injection or transplantation site, and the need to ensure continuous effect of therapy and appropriate timing with relation to when to intervene in the course of disease.

Finally, the main obstacles remaining include our inability to distinguish between primary and secondary disease mechanisms. This raises the question as to which of the many cellular pathways of pathogenesis would be the most effective target in influencing disease onset and progression. Further identification of abnormalities, pathways, and targets that are the most critical for neurodegeneration and discriminating them from the ones that are secondary responses or just related phenomena are required.
